# Efficient and Robust *Paramyxoviridae* Reverse Genetics Systems

**DOI:** 10.1128/mSphere.00376-16

**Published:** 2017-03-29

**Authors:** Shannon M. Beaty, Arnold Park, Sohui T. Won, Patrick Hong, Michael Lyons, Frederic Vigant, Alexander N. Freiberg, Benjamin R. tenOever, W. Paul Duprex, Benhur Lee

**Affiliations:** a Department of Microbiology, Icahn School of Medicine at Mount Sinai, New York, New York, USA; b Department of Microbiology, Immunology, and Molecular Genetics, University of California Los Angeles, Los Angeles, California, USA; c Department of Pathology, University of Texas Medical Branch, Galveston, Texas, USA; d Department of Microbiology, Boston University School of Medicine, Boston, Massachusetts, USA; CDC

**Keywords:** paramyxovirus, reverse genetic analysis, ribozymes

## Abstract

The notoriously low efficiency of *Paramyxoviridae* reverse genetics systems has posed a limiting barrier to the study of viruses in this family. Previous approaches to reverse genetics have utilized a wide variety of techniques to overcome the technical hurdles. Although robustness (i.e., the number of attempts that result in successful rescue) has been improved in some systems with the use of stable cell lines, the efficiency of rescue (i.e., the proportion of transfected cells that yield at least one successful rescue event) has remained low. We have substantially increased rescue efficiency for representative viruses from all five major *Paramyxoviridae* genera (from ~1 in 10^6^-10^7^ to ~1 in 10^2^-10^3^ transfected cells) by the addition of a self-cleaving hammerhead ribozyme (Hh-Rbz) sequence immediately preceding the start of the recombinant viral antigenome and the use of a codon-optimized T7 polymerase (T7opt) gene to drive paramyxovirus rescue. Here, we report a strategy for robust, reliable, and high-efficiency rescue of paramyxovirus reverse genetics systems, featuring several major improvements: (i) a vaccinia virus-free method, (ii) freedom to use any transfectable cell type for viral rescue, (iii) a single-step transfection protocol, and (iv) use of the optimal T7 promoter sequence for high transcription levels from the antigenomic plasmid without incorporation of nontemplated G residues. The robustness of our T7opt-HhRbz system also allows for greater latitude in the ratios of transfected accessory plasmids used that result in successful rescue. Thus, our system may facilitate the rescue and interrogation of the increasing number of emerging paramyxoviruses.

**IMPORTANCE** The ability to manipulate the genome of paramyxoviruses and evaluate the effects of these changes at the phenotypic level is a powerful tool for the investigation of specific aspects of the viral life cycle and viral pathogenesis. However, reverse genetics systems for paramyxoviruses are notoriously inefficient, when successful. The ability to efficiently and robustly rescue paramyxovirus reverse genetics systems can be used to answer basic questions about the biology of paramyxoviruses, as well as to facilitate the considerable translational efforts being devoted to developing live attenuated paramyxovirus vaccine vectors.

## Abstract (precis)

The ability to manipulate the genome of paramyxoviruses and evaluate the effects of these changes at the phenotypic level is a powerful tool for the investigation of specific aspects of the viral life cycle and viral pathogenesis. However, reverse genetics systems for paramyxoviruses are notoriously inefficient, when successful. The ability to efficiently and robustly rescue paramyxovirus reverse genetics systems can be used to answer basic questions about the biology of paramyxoviruses, as well as to facilitate the considerable translational efforts being devoted to developing live attenuated paramyxovirus vaccine vectors.

## INTRODUCTION

The *Paramyxoviridae* family, which belongs to the order *Mononegavirales*, is comprised of enveloped viruses that have a nonsegmented, negative-sense single-stranded RNA genome. The family includes many viruses that have a significant impact on human health and agriculture, such as measles virus (MeV), mumps virus (MuV), human parainfluenza virus types 1 to 4 (hPIV1 to -4), Newcastle disease virus (NDV), Nipah virus (NiV), and Hendra virus (HeV). The first successful full-length paramyxovirus reverse genetics systems—which drive the *de novo* production of replication-competent viruses from a cDNA template encoding the viral genome—were developed in parallel by three different research groups in 1995, and recombinant viruses have since been recovered from all major genera within the family (1–6). However, virus rescue (i.e., recovery of infectious viral particles) remains inefficient and nonrobust for many paramyxovirus reverse genetics systems; successful virus production typically requires large numbers of transfected cells and repeated rescue attempts. There are many unique challenges that contribute to the inefficiency of virus rescue for paramyxoviruses. Historical perspectives on paramyxovirus reverse genetics systems have been published by Conzelmann (2004) (7) and Pfaller et al*.* (2015) (8), and specific challenges to virus rescue are discussed in detail here.

Reverse genetics can be used to produce modified viruses that have specific properties with potential applications in vaccine development, gene therapy, and basic science. The lack of efficient and reliable systems that could allow for genetic manipulation of paramyxovirus genomes has hindered development of vaccines and antiviral drugs and has limited the kind of experiments that can be done to interrogate the basic biology of these viruses, such as the rescue and characterization of highly attenuated mutants or mutational libraries. We have developed highly efficient reverse genetics systems for a broad range of paramyxoviruses spanning all major genera. Here, we present a strategy to efficiently recover recombinant virus that does not require the use of a helper virus (e.g., vaccinia virus-T7) or stable T7-expressing cell lines. Our method is robust, reliable, and has high potential for flexibility in the cell type and the ratio of plasmids used for rescue.

## RESULTS

**FIG 1:**
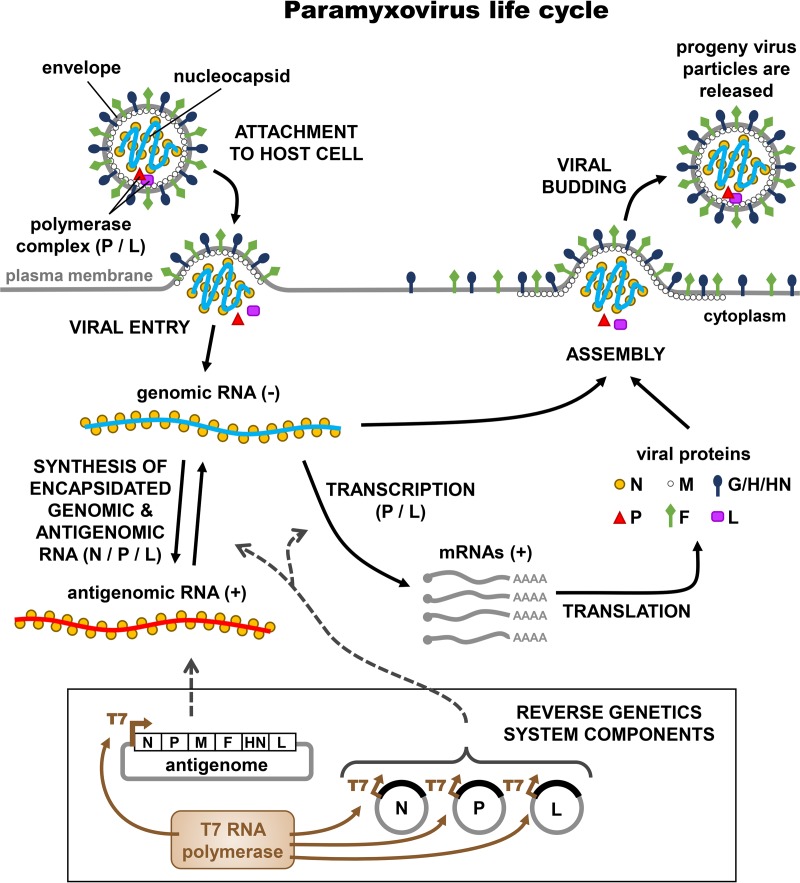
Components of a paramyxovirus reverse genetics system and their relationships to the viral life cycle. The schematic illustrates the major steps of the viral life cycle. The inset box shows the components of a paramyxovirus reverse genetics system, with dashed gray arrows indicating their participation in various steps of the viral life cycle.

In contrast with positive-strand RNA viruses and most DNA viruses, introduction of genomic material from negative-sense RNA viruses into an appropriate host cell is not sufficient to initiate an infectious life cycle (7). A generalized schematic of the paramyxovirus life cycle and its relationship to virus rescue is illustrated in Fig. 1. The minimal unit for initiating replication in paramyxoviruses is the ribonucleoprotein (RNP) complex, in which the RNA genome or antigenome is encapsidated by the viral nucleocapsid (N or NP) and further associated with the viral RNA-dependent RNA polymerase (RdRp) complex. Transcription and replication of genomic or antigenomic RNA must be mediated by the corresponding RdRp complex, which is comprised of the phosphoprotein (P) and large protein (L) (7). As a result of these constraints, rescue requires the intracellular transcription of full-length genomic (or antigenomic) RNA and simultaneous expression of the viral proteins (N, P, and L) that form the RNP complex. Typically, transcription of the viral antigenome—as well as that of the N, P, and L support plasmids—is driven by T7 polymerase, a bacteriophage DNA-dependent RNA polymerase that predominantly localizes to the cytoplasm of eukaryotic cells when expressed ectopically. Since transcription of all four plasmids of a typical paramyxovirus reverse genetics system is driven by T7 polymerase, the efficiency of viral rescue is dependent on the efficient delivery and expression of the polymerase. During the course of developing reverse genetics technologies, many different strategies have been used to deliver T7 polymerase intracellularly during rescue. Table 1 details the rescue efficiencies of different approaches that have been utilized by reverse genetics systems for paramyxoviruses and other members of the *Mononegavirales* order.

**TABLE 1:** Approaches and rescue efficiencies of reverse genetics systems for paramyxoviruses and other selected nonsegmented negative-sense RNA viruses^a^

Family, genus, and/or virus	Nontemplated Gs in T7 promoter	T7 delivery method	Rescue cell type	Rescue efficiency (no. of events/10^5^ cells)	Reference
*Paramyxoviridae*					
*Respirovirus*					
SeV	3G	vTF7-3	BHK or HeLa	NR	2
SeV	No G	T7-vaccinia virus (vTF7-3)	LLCMK2	1	33
**SeV**	**3G-Hh-Rbz**	**T7opt**	**BSR-T7/5**	**~4,260**	**This study**
HPIV-1	2G	T7-vaccinia virus (MVA-T7)	HEp-2	NR	34
BPIV3	2G	T7-vaccinia virus (MVA-T7)	HEp-2	NR	35
HPIV-3	2G	T7-vaccinia virus (vTF7-3)	HeLa	NR	36
HPIV-3	No G and 2G	T7-vaccinia virus (MVA-T7)	HEp-2	NR	37
**HPIV-3**	**3G-Hh-Rbz**	**T7opt**	**BSR-T7/5**	**~600**	**This study**
*Morbillivirus*					
RPV	No G and 2G	T7-vaccinia virus (MVA-T7)	293	0.1	38
CDV	No G	T7-vaccinia virus (MVA-T7)	HeLa	0.1–0.2	39
MeV	No G	293 cells stably expressing T7 and MeV-N/P/L	293-3-46	0.1–0.6	1
**MeV**	**3G-Hh-Rbz**	**T7opt**	**BSR-T7/5**	**~300**	**This study**
*Rubulavirus*					
HPIV-2	3G	T7-vaccinia virus (MVA-T7)	Vero	0.5	40
SV5	3G	T7-vaccinia virus (MVA-T7)	A549	>0.01 (estimated)	4
MuV	No G	T7-vaccinia virus (MVA-T7)	A549	0.03–0.2	41
**MuV**	**3G-Hh-Rbz**	**T7opt**	**BSR-T7/5**	**~1,000**	**This study**
*Avulavirus*					
NDV	3G	BSR-T7/5	BSR-T7/5	NR	5
NDV	2G	T7-fowlpox virus (FPV-T7)	CEF or QM5	0.5–5	42
NDV	3G-Hh-Rbz, CMV-Hh-Rbz	BSR-T7/5, RNA Pol II	CEF, BHK-21, BSR-T7/5	NR	43
**NDV**	**3G-Hh-Rbz**	**T7opt**	**BSR-T7**	**~440**	**This study**
*Henipavirus*					
HeV	No G	pCAGGS T7 plasmid (wild-type T7 from BSR-T7/5 cells)	293T	NR	20
NiV	No G	T7-vaccinia virus (MVAGKT7)	CV-1	NR	6
**NiV**	**3G-Hh-Rbz**	**T7opt**	**BSR-T7/5**	**~10**	**This study**
*Pneumoviridae*					
BRSV	3G	BSR-T7/5 (creator of this line)	BSR-T7/5	~1	9
HRSV	3G	T7-vaccinia virus (MVA-T7)	Hep-2	0.7–6.3	3
HRSV	3G	T7-vaccinia virus (MVA-T7)	HEp-2	0.7–6.7	44
*Rhabdoviridae*					
RV	3G	T7-vaccinia virus (vTF7-3)	BHK-21 (clone BSR)	0.01	45
VSV	3G	T7-vaccinia virus (vTF7-3)	BHK-21	0.001–0.02	46
VSV	2G	T7-vaccinia virus (vTF7-3)	BHK stably expressing VSV-NP	NR	47
*Filoviridae*					
EBOV	1G and 2G	BSR-T7/5	BSR-T7/5	~3	48

a Reported or calculated measurements of rescue efficiencies, using the indicated approach to virus rescue. Data reported in boldface were obtained in this study. NR, not reported; passaging the virus was required for detection of infectious virus and rescue efficiency could not be calculated. Abbreviations (those not defined in text): RPV, Rinderpest virus; CDV, canine distemper virus; SV5, Simian virus 5; bRSV, bovine respiratory syncytial virus; hRSV, human respiratory syncytial virus; RV, rabies virus; VSV, vesicular stomatitis virus; EBOV, Ebola virus; CMV, cytomegalovirus; RNA Pol II, polymerase II.

### Effect of codon optimization of the T7 RNA polymerase gene.

**FIG 2:**
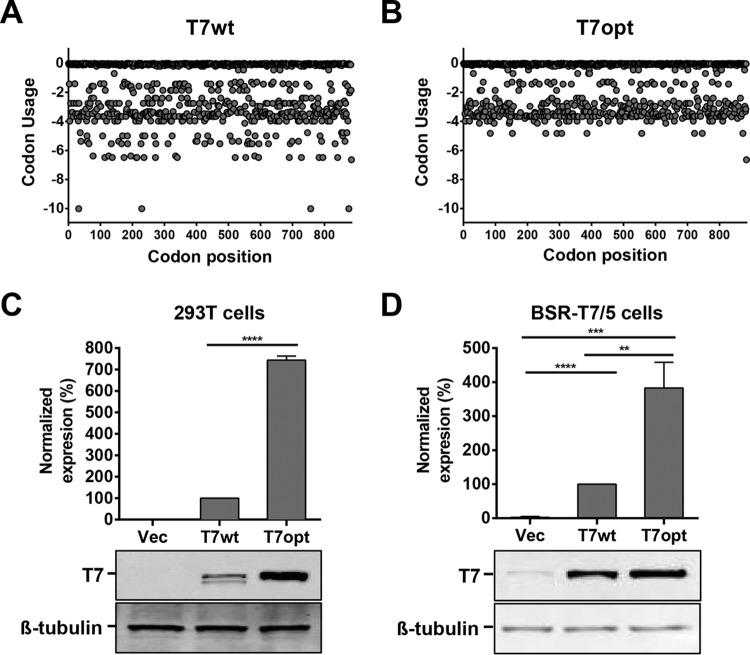
Codon optimization of the T7 gene enhances protein expression. (A and B) Human codon usage of the T7wt gene (A) and T7opt gene (B) was calculated using the JCat codon usage calculator published by Grote et al. in 2005 (32). (C and D) Protein expression of the T7 polymerase was evaluated in HEK293T (C) and BSR-T7/5 (D) cells by Western blotting densitometry. Blots were stained with mouse anti-T7 and rabbit anti-β-tubulin primary antibodies. T7 polymerase expression was normalized to β-tubulin expression, and the expression of T7wt was set to 100%. Bars show the mean densitometry quantification for three biological replicates, and error bars indicate 1 standard deviation. Statistical significance was evaluated by using a two-tailed unpaired *t* test. **, *P* < 0.01; ***, *P* < 0.001; ****, *P* < 0.0001.

To achieve high levels of cytoplasmic T7 polymerase without the use of vaccinia virus, we first codon optimized the T7 gene, which was derived from a bacteriophage and displays suboptimal codon usage when expressed in mammalian cells. Thus, we generated a T7 polymerase gene that is codon optimized for expression in mammalian cells (T7opt) (Fig. 2A and B) and cloned it into a pCAGGS expression vector, which was designed to drive high levels of gene expression. The T7 polymerase expression levels in cells transfected with T7opt were evaluated by Western blotting (Fig. 2C and D), and were significantly higher than that in cells transfected with a homologous plasmid containing the wild-type T7 gene (T7wt), both in HEK293T cells and BSR-T7/5 cells. BSR-T7/5 cells are a BHK-derived stable T7 cell line created by Buchholz et al. in 1999 and are commonly used for paramyxovirus reverse genetics (9). Codon optimization of the T7 gene significantly enhances expression of the polymerase. Furthermore, supplementation of BSR-T7/5 cells with T7 polymerase from either T7wt or T7opt significantly enhanced T7 polymerase expression over endogenous levels, with T7opt conferring an ~150-fold increase over endogenous T7 expression levels (Fig. 2D).

**FIG 3:**
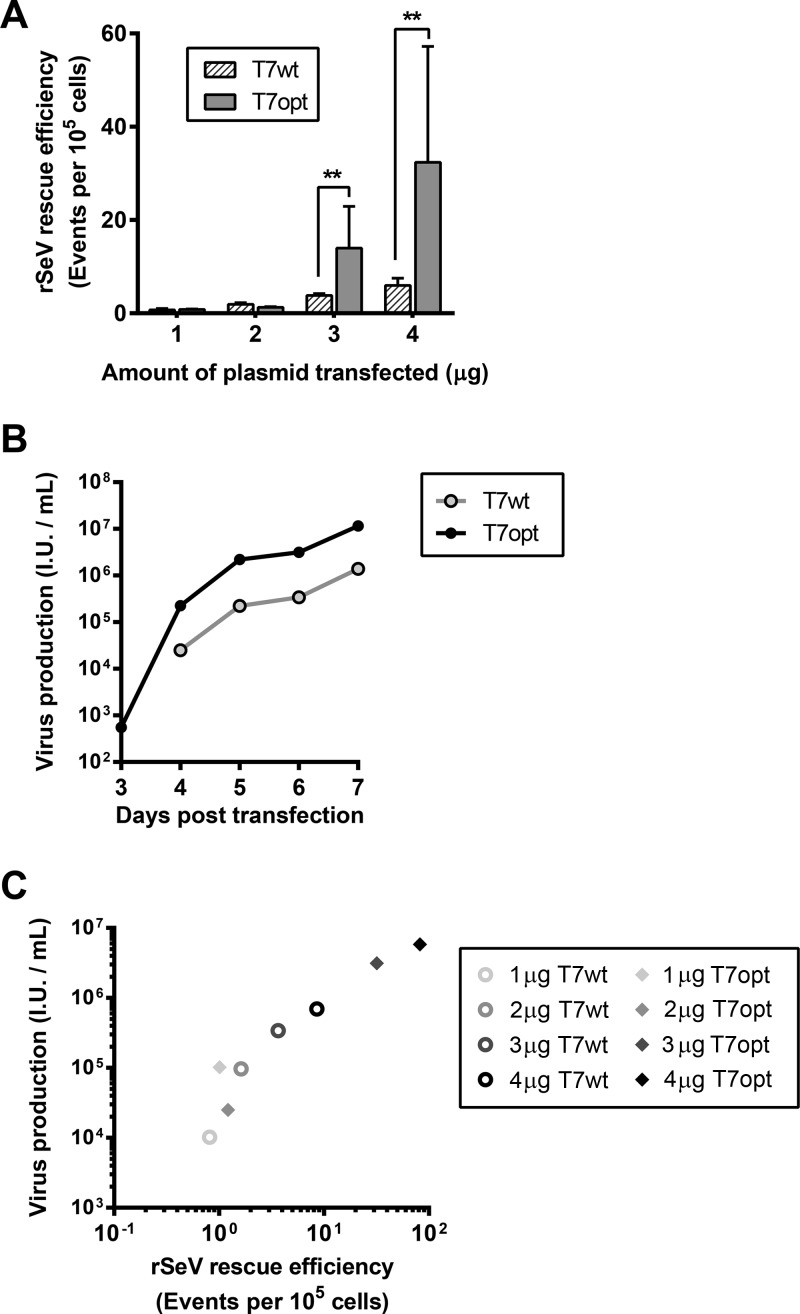
Codon-optimized T7 polymerase increases rescue efficiency and virus production. (A) Efficiency of rSeV^Fushimi^-EGFP rescue on HEK293T cells was quantified by fluorescence-activated cell sorting at 2 days posttransfection. Statistical significance was evaluated by using a two-tailed unpaired *t* test. **, *P* < 0.01. (B) rSeV^Fushimi^-EGFP produced by rescue on HEK293T cells using 3 μg T7opt was quantified by determination of titers on Vero cells. (C) Rescue efficiency was plotted against the titers corresponding to rescue at 6 days posttransfection. Pearson’s correlation was used to evaluate the significance of the relationship (Pearson’s *r* = 0.9899, *P* < 0.0001).

In order to evaluate the effect of T7 codon optimization on virus rescue, either T7opt or T7wt was used to drive rescue of a recombinant Sendai virus with an enhanced green fluorescent protein (EGFP) reporter (rSeV^Fushimi^-EGFP) in HEK293T cells, using a single-step transfection protocol described in Materials and Methods. The use of T7opt significantly increased the frequency of GFP-positive rescue cells (i.e., rescue efficiency), rSeV^Fushimi^-EGFP, above that with rescue driven by T7wt (Fig. 3A). The titer of rSeV^Fushimi^-EGFP produced in the rescue well was similarly enhanced for rescue driven by T7opt compared to rescue with T7wt (Fig. 3B). GFP-positive rescue events were highly correlated with virus production (Fig. 3C; Pearson’s *r* = 0.9899, *P* < 0.0001); this finding supports the biological relevance of early quantification of GFP-positive rescue cells as a metric for rescue efficiency.

### Introduction of a hammerhead ribozyme sequence into the viral antigenomic plasmid.

It has been shown *in vitro* that the inclusion of three guanine (G) residues at the 3′-end of the T7 promoter enhances processivity of the T7 RNA polymerase and dramatically increases transcription levels (10). However, in the context of paramyxovirus reverse genetics systems, the use of the optimal (3G) T7 promoter has mixed effects on virus rescue. Although use of the optimal promoter to drive rescue does provide an advantage at the level of transcription, the extra Gs are positioned after the transcriptional start site and thus are incorporated into the 5′-end of the viral antigenomic transcript. These extra nucleotides not only interfere with appropriate recognition of the genomic terminus by the RdRp complex, but also conflict with the “rule of six,” a property shared by all members of the *Paramyxoviridae* family, which is based on the requirement that the genome be an exact multiple of 6 nucleotides in length for robust virus rescue and efficient viral replication (11, 12). Approaches to mitigating this conflict are varied in paramyxovirus reverse genetics systems; in some cases, the “no G” approach yields the highest rescue efficiency, and in other cases the addition of one, two, or three extra Gs is advantageous or necessary for successful rescue (Table 1).

**FIG 4:**
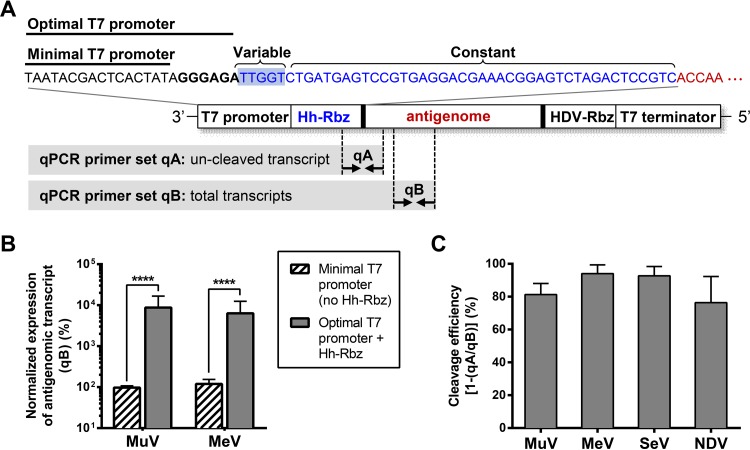
Introduction of an autocatalytic hammerhead ribozyme sequence permits use of the optimal T7 promoter to enhance transcription levels. (A) Sequence of the Hh-Rbz (blue text) and its context within the plasmid carrying the viral antigenome (red text). The variable region is the reverse complement of the start of the antigenome, while the constant region is fixed, regardless of the sequence context. The sequence of the minimal and optimal T7 promoter is shown in black text. Dashed lines indicate the approximate primer binding regions of the qPCR primers used in panels B and C. (B) Transcription levels measured by qPCR were normalized to the minimal T7 promoter (no Hh-Rbz) condition. Bars represent the means of four replicates, and error bars indicate 1 standard deviation. Significance was evaluated by using a two-tailed unpaired *t* test. ****, *P* < 0.0001. (C) Quantification of cleavage efficiency measured by qPCR. Bars represent the means of four replicates, and error bars indicate 1 standard deviation.

To overcome the low rescue efficiency that results from the use of a suboptimal T7 promoter or the incorporation of extra nonviral residues at the 5′-end of the antigenome, we introduced an autocatalytic hammerhead ribozyme sequence (Hh-Rbz) between the optimal T7 promoter and the start of the antigenome (Fig. 4A). A similar Hh-Rbz design has been used by various groups to rescue viruses of the *Pneumoviridae* and *Rhabdoviridae* families, but this strategy has not been widely used for paramyxovirus rescue (13–16). For paramyxoviruses, this Hh-Rbz ensures adherence to the “rule of six” by self-cleaving immediately preceding the start of the viral antigenomic transcript; thus, Hh-Rba leaves the native 5′ antigenomic terminus intact. The effectiveness of various hammerhead ribozyme sequences was tested in two ways: by a quantitative PCR (qPCR)-based cleavage assay using a recombinant NiV (rNiV) minigenome construct and by comparison of rescue efficiency in the context of a full-length rNiV antigenomic construct, as described by Yun et al. (17). For rMuV, rMeV, rSeV, and rNDV, transcription levels of the antigenome and Hh-Rbz cleavage efficiencies were quantified by using qPCR, with the proportion of uncleaved and total transcripts measured in RNA isolated from BSR-T7/5 cells that had been transfected with the indicated antigenomic construct. While the rSeV^Fushimi^-EGFP and rNDV^LaSota^-EGFP constructs already had the optimal T7 promoter before we introduced modifications, the rMuV^JL5^-EGFP and rMeV^EdmonstonB^-EGFP constructs only had the minimal T7 promoter. We found that use of the optimal T7 promoter for rMuV^JL5^-EGFP and rMeV^EdmonstonB^-EGFP significantly enhanced transcription levels, with an increase of ~2 logs over that of homologous constructs containing the minimal T7 promoter and no Hh-Rbz (Fig. 4A and B). Hh-Rbz-mediated cleavage of the RNA transcript was highly efficient, ranging from 76 to 94% in the four paramyxovirus constructs tested in this study (Fig. 4C). For full transparency and reproducibility, the optimized version of all antigenomic plasmids and support plasmids have been sequenced and deposited in GenBank (accession numbers KY295909 to KY295932).

### High rescue efficiency results from the combined use of T7opt and a hammerhead ribozyme.

**FIG 5:**
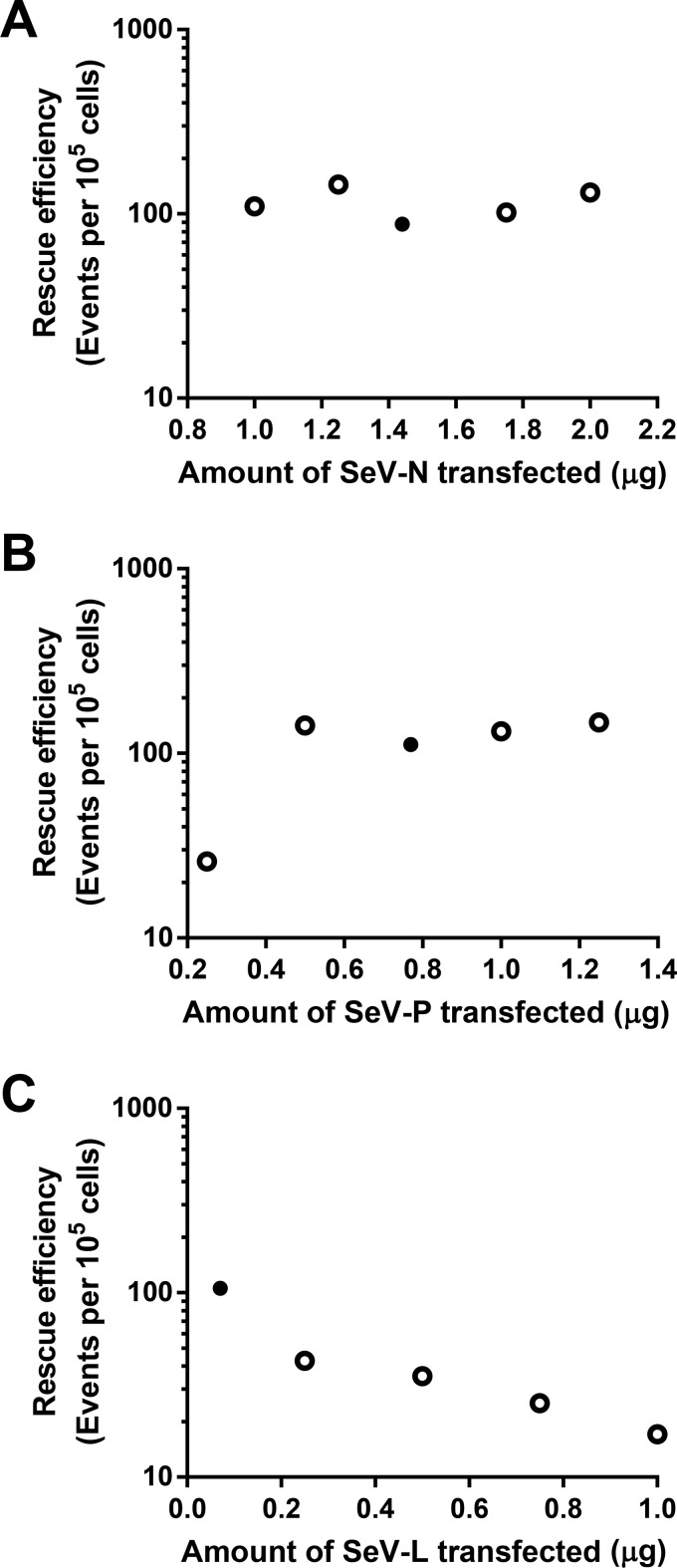
Use of T7opt and Hh-Rbz permits flexibility in the amount of support plasmids transfected. Rescue transfections were performed on HEK293T cells. Rescue efficiency was quantified by fluorescence-activated cell sorting of the rescue cells at 2 days posttransfection to determine the frequency of EGFP-positive cells. Filled points indicate the standard amount of the indicated plasmid that was used in all other rSeV^Fushimi^-EGFP rescue transfections in this study.

We tested the flexibility of our optimized rSeV^Fushimi^-EGFP reverse genetics system by varying the amount of N, P, and L support plasmids used for rescue. Typically, the paramyxovirus support proteins must be expressed in a precise ratio for successful virus rescue (7). The absolute amounts and ratios of transfected plasmids vary considerably in different paramyxovirus reverse genetics systems, but generally, N is needed in the most abundant amounts, followed by P, then L, which reflects the relative abundance of these proteins during infection. Although the amount of SeV-N, SeV-P, and SeV-L plasmid used for rSeV^Fushimi^-EGFP rescue covered a very broad range (~1 μg), every rescue attempt was successful, albeit with varying efficiencies (Fig. 5A to C). The Hh-Rbz-T7opt rescue approach allows for significant latitude in the ratios of transfected plasmids, and rescue efficiency was consistently high in every rescue attempt.

**FIG 6:**
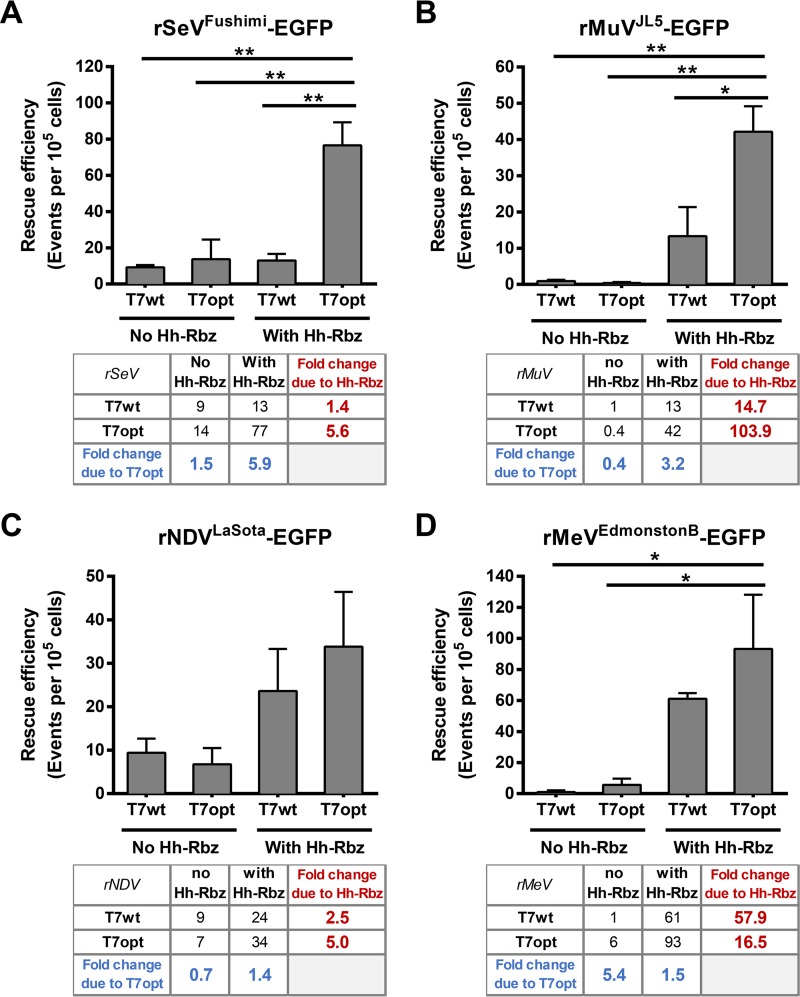
Combined use of T7opt and Hh-Rbz synergistically enhances rescue efficiency of paramyxovirus reverse genetics systems. Rescue efficiency of reverse genetics systems for rSeV (*Respirovirus*) (A), rMuV (*Rubulavirus*) (B), rNDV (*Avulavirus*) (C), and rMeV (*Morbillivirus*) (D) on HEK293T cells. For rMuV and rMeV, the premodified constructs lacked an optimal T7 promoter, and the addition of the Hh-Rbz therefore also included the addition of the optimal promoter. Bars show the means of three replicates, and error bars indicate the standard errors of the means. The inset shows the actual *y* axis numbers under the indicated conditions and the fold change in rescue efficiency that was due to the introduction of the Hh-Rbz (red text) or the use of T7opt (blue text). Statistical significance was evaluated by a one-way analysis of variance with a Bonferroni correction. *, *P* < 0.05; **, *P* < 0.01.

The combined use of T7opt and an Hh-Rbz was employed to rescue representative viruses from four of the five major *Paramyxoviridae* genera, and rescue efficiencies were compared with those of homologous antigenomic constructs lacking the Hh-Rbz and with the use of T7wt (Fig. 6A to D). The T7opt-Hh-Rbz approach yielded a high rescue efficiency for all four reverse genetics systems. The effect of the Hh-Rbz appears to involve a stronger contribution to increasing rescue efficiency than with use of T7opt, as indicated by the higher fold change due to the presence of the Hh-Rbz in all cases except rSeV. The combined use of an Hh-Rbz sequence before the start of the viral antigenome and T7opt to drive rescue can have an additive effect to dramatically increase the robustness, efficiency, and flexibility of paramyxovirus reverse genetics systems. Using the approach described in this study, we have also constructed a reverse genetics system for human parainfluenza virus type 3 (HPIV-3) *de novo* from synthetic DNA. HPIV-3 is an important human pathogen in the *Respirovirus* genus. We used the same amounts of plasmids and transfection reagents as in the rSeV^Fushimi^-EGFP reverse genetics system, with the hope that the flexibility we observed with rSeV^Fushimi^-EGFP (Fig. 5A to C) would provide some latitude in the conditions for successful rescue of rHPIV3^JS^-G.Luc-EGFP without onerous reoptimization. Indeed, we found that use of the parameters from the SeV rescue system resulted in high rescue efficiency for rHPIV3^JS^-G.Luc-EGFP (~600 events per 10^5^ transfected cells) (Table 1).

## DISCUSSION

A recombinant T7-expressing vaccinia virus helper is commonly used in reverse genetics systems to drive high-level expression of T7 polymerase in the cytoplasm. However, the use of vaccinia virus has several disadvantages, primarily high poxvirus-mediated cytotoxicity, challenges associated with separating vaccinia virus from the rescued paramyxovirus, and increasing restrictions on the use of vaccinia virus, especially for paramyxovirus vaccine development. Due to these complications, cell lines stably expressing T7 polymerase have been developed as alternatives to the use of a helper T7-vaccinia virus. Several different stable T7 cell lines have been developed to date, the most commonly used of which are BSR-T7/5 cells, a BHK-derived cell line created by Buchholz et al. (9). Stable T7-expressing cell lines effectively circumvent many of the issues associated with the use of vaccinia virus; however, with the possible exception of MeV rescue, none has found broad acceptance as a substitute for the use of T7-vaccinia virus. This is largely due to the fact that stable T7 cell lines have significantly lower T7 expression levels than those of vaccinia virus-mediated systems (18), and T7 expression has been shown to be a major determinant of rescue efficiency (1). While the reason for this lower expression of T7 in stable cells has not been definitively explained, it might be the result of suboptimal codon usage in the bacteriophage-derived T7 gene or unwanted splicing that might occur at the mRNA level when the T7 gene is expressed from the nucleus. Furthermore, the use of stable cell lines imposes an experimental limitation, as it inherently constrains the cell type that can be used for rescue.

Virus rescue experiments were performed on HEK293T cells, which do not endogenously express T7 polymerase; thus, the use of T7opt makes efficient rescue of paramyxoviruses possible without the use of vaccinia virus or stable T7 cell lines. It should be possible to adapt our one-step all-plasmid transfection protocol to rescue recombinant paramyxoviruses on cell lines such as the WHO seed stocks of Vero cells approved for use in human clinical trials. A plasmid-encoded T7 polymerase has previously been used to drive successful rescue of HeV and Crimean Zaire hemorrhagic fever virus (CCHFV); in the latter case, codon optimization of the viral RdRp L gene was critical for efficient rescue (19, 20). A plasmid-based method for delivering T7 polymerase is advantageous because it theoretically allows for the use of any transfectable cell type. Therefore, using this system would allow the cell type to be selected with greater consideration for the tropism of the virus or the experimental design.

Another factor that significantly contributes to the inefficiency of paramyxovirus rescue is the requirement for an exact end-to-end copy of the viral genome, which can be difficult to achieve with traditional reverse genetics systems. The genome length must also comply with the “rule of six,” in which the total number of nucleotides must be an exact multiple of six for the virus to replicate efficiently. Tolerance for variation is particularly constrained in the genomic termini, as they contain critical elements for replication, such as the signal for encapsidation and promoters for genome and antigenome replication. In standard reverse genetics systems, an exact 3′-end of the antigenome is generated by autocatalytic cleavage from a hepatitis delta virus ribozyme (HDV-Rbz) sequence positioned directly downstream of the antigenome (5′-GGCCGGCATGGTCCCAGCCTCCTCGCTGGCGCCGGCTGGGCAACATTCCGAGGGGACCGTCCCCTCGGTAATGGCGAATGGGAC-3′). Creating a precise copy of the 5′-end of the antigenome, however, has proven more challenging. While both genomic termini have poor tolerance for the addition of nontemplated nucleotides, the 5′-end of the antigenome may be particularly constrained. For vesicular stomatitis virus, a related member of the *Mononegavirales* order, genomic constructs with short heterologous extensions were tolerated on the 5′-end (3′-end of the antigenome), but extra nonviral residues on the genomic 3′-terminus prevented replication (21). Moreover, the first several nucleotides of the paramyxovirus genome are highly conserved and start with the sequence 5′-ACCAA-3′ (antigenomic sense), almost without exception (22). Thus, the viral requirement for a 5′-ACCAA-3′ transcriptional start sequence is in direct conflict with the T7 polymerase requirement for a 5′-GGG-3′ transcriptional start sequence for optimal processivity.

Rescue efficiency of a rabies virus minigenome was significantly improved by the use of an Hh-Rbz inserted between the T7 promoter and the start of the minigenome, which allowed for the 3G optimal promoter without adding nonviral nucleotides to the minigenome (23). This strategy was applied to a full-length rabies virus antigenome, which increased rescue efficiency 100-fold when used in combination with an enhanced HDV-Rbz (16), but rescue efficiency was surprisingly poor when an Hh-Rbz approach was used to rescue Borna disease virus and measles virus (24). This difference in effectiveness of the Hh-Rbz in these studies might be attributed to variation in the cleavage efficiency of the different ribozyme sequences used, as Hh-Rbz sequences are a diverse family of endonucleolytic ribozymes found in organisms spanning almost every kingdom of life and cleavage efficiency can vary considerably among distinct sequences (25). Additionally, the effectiveness of the Hh-Rbz approach might have also been reduced by constraints that limit the expression of T7 RNA polymerase, as discussed above.

Reverse genetics systems for paramyxoviruses are notoriously inefficient and, when successful, often yield inconsistent results. We have substantially increased rescue efficiency and robustness for a diverse panel of paramyxoviruses, and this technological advance allowed for the first genome-wide mutagenesis study of a paramyxovirus, reported by Fulton et al. in 2015 (26), which was performed using the MeV reverse genetics system that is described in the present study. We have also used the highly efficient design described in the present study to construct second-generation reverse genetics systems for NiV and HeV, as described in detail by Yun et al*.* (17). Taken together, this approach to paramyxovirus rescue appears to be generalizable across the entire *Paramyxoviridae* family and has been effective in every system we have built or modified to date. Our T7opt-Hh-Rbz methodology allows for faster, safer, and more efficient viral rescue, which can be leveraged to functionally interrogate paramyxovirus biology on a genomic level and broaden the range of tools available to vaccinologists.

## MATERIALS AND METHODS

### Cell lines.

HEK293T cells, Vero cells (CCL-81; ATCC), and BSR-T7/5 cells (9) were propagated in Dulbecco’s modified Eagle’s medium (Invitrogen) supplemented with 10% fetal bovine serum (FBS; Atlanta Biologicals) and penicillin-streptomycin at 37°C. BSR-T7/5 cells were additionally cultured in medium containing 1 mg/ml G418 to maintain the T7 polymerase transgene.

### Paramyxovirus reverse genetics plasmids.

All modifications to reverse genetics plasmids were performed via insertion of modified PCR fragments into constructs at unique restriction sites via ligation-independent In-Fusion (Clontech). The modified fragments were generated via standard and overlapping PCR, using Velocity DNA polymerase (Bioline). Full-length paramyxovirus antigenomic constructs were maintained in Stbl2 *Escherichia coli* (Invitrogen) with growth at 30°C. The wild-type bacteriophage T7 sequence was codon optimized (GenScript) and inserted into pCAGGS (T7opt; deposited in Addgene as plasmid 65974).

### i. Sendai virus plasmids.

The rSeV^Fushimi^-EGFP clone was derived from SeV Fushimi strain with mutations introduced into the F and M genes to allow trypsin-independent growth, as previously described by Hou et al. (27). The rSeV reverse genetics system was a generous gift from Nancy L. McQueen. An EGFP reporter gene was inserted as an additional reading frame between the N and P genes via duplication of the N-P intergenic region, as described previously (28). The Hh-Rbz sequence was inserted between the T7 promoter and the start of the viral antigenome. T7-driven SeV^Fushimi^-N, SeV^Fushimi^-P, and SeV^Fushimi^-L were as described by Hou et al*.* (27).

### ii. Mumps virus plasmids.

The rMuV^JL5^-EGFP clone was based on the Jeryl Lynn 5 (JL5) vaccine strain, as described by Lemon et al*.* (29). The rMuV antigenomic plasmid was a generous gift from W. Paul Duprex. A reporter gene encoding EGFP was inserted as an additional reading frame between the N and P reading frames via duplication of the N-P intergenic region. The Hh-Rbz sequence was inserted between the T7 promoter and the start of the viral antigenome, and the T7 promoter was modified to the optimal sequence.

### iii. Newcastle disease virus plasmids.

The rNDV^LaSota^-EGFP clone was based on the LaSota strain, as described by Elankumaran et al. (30), which contains an EGFP reporter gene as an additional reading frame between the P and M reading frames via duplication of the N-P intergenic region. The rNDV reverse genetics system was a generous gift from Subbiah Elankumaran. The native cleavage site for proteolytic processing of F was replaced with the cleavage site of urokinase-type plasminogen activator (uPA) to allow for trypsin-independent replication in tissue culture. The Hh-Rbz sequence was inserted between the T7 promoter and the start of the viral antigenome.

### iv. Measles virus plasmids.

The rMeV^EdmonstonB^-EGFP clone was derived from the Edmonston B strain, as described by Duprex et al. (31). The Hh-Rbz sequence was inserted between the T7 promoter and the start of the viral antigenome, and the T7 promoter was modified to the optimal sequence. T7-driven MeV-N, -P, and -L support plasmids were contributed by Richard Plemper and carried the genes cloned from a primary isolate of MeV.

### v. Human parainfluenza virus type 3 plasmids.

The rHPIV3^JS^-G.luc-EGFP clone was constructed entirely from synthetic DNA using the antigenomic sequence of the JS strain preceded by the optimal T7 promoter and the Hh-Rbz sequence. The HDV ribozyme and two tandem T7 terminators were placed downstream of the antigenome, and the entire antigenomic cassette was inserted into a pTM1 vector backbone. HPIV3^JS^-N, -P, and -L support plasmids were cloned out of the antigenome and inserted into a pTM1 vector backbone under control of a T7 promoter.

### vi. Nipah virus plasmids.

The rNiV^Malaysia^-EGFP clone and the T7-driven NiV^Malaysia^-N, -P, and -L support plasmids were based on the prototype Malaysia strain and were previously described by Yun et al. (17). The rNiV antigenomic plasmid has the Hh-Rbz sequence inserted between the optimal T7 promoter and the start of the viral antigenome.

### Immunoblotting.

For comparison of T7 polymerase protein expression upon transfection of T7wt or T7opt, HEK293T or BSR-T7/5 cells were transfected in a 12-well format with 0.5 μg T7wt, T7opt, or empty pCAGGS vector using Lipofectamine LTX with PLUS reagent (Invitrogen) according to the manufacturer’s protocol. At 24 h posttransfection, cell lysates were collected in SDS Laemmli buffer, boiled, and run on 10% Tris-glycine SDS-PAGE gels. Upon transfer to polyvinylidene difluoride membranes (Immobilon-F; Millipore), the membranes were blocked in Odyssey blocking buffer (LI-COR Biosciences), then incubated with a mouse anti-T7 antibody (catalog number 69522; Millipore) and a rabbit anti-β-tubulin antibody (clone 9F3; catalog number 2128; Cell Signaling Technology, Inc.), followed by fluorescent IRDye 800CW and 680LT antibodies (LI-COR Biosciences). Images were obtained on a LI-COR Odyssey imaging system, and densitometry of protein bands was quantified using the LI-COR Odyssey software.

### Recovery of recombinant viruses from cDNA.

For virus rescue, 1 × 10^6^ HEK293T cells or 4 × 10^5^ BSR-T7/5 cells per well were seeded in each well of a 6-well plate in order to achieve ~50% confluence on the day of transfection. The following day, the indicated amounts (detailed below) of antigenomic construct, N, P, and L support plasmids, a plasmid encoding T7 polymerase, and Lipofectamine LTX/PLUS transfection reagent (Invitrogen) were combined in 200 μl Opti-MEM (Invitrogen) and mixed by pipetting gently. T7opt or T7wt was included in the rescue transfection mixture for all cell types, including BSR-T7/5 cells. After incubation at room temperature for 30 min, the DNA-Lipofectamine mixture was added dropwise onto cells. (It is critical that the DNA-Lipofectamine mixture not be mixed before adding to cells, as mixing can disrupt the liposomes.) Transfected cells were incubated at 37°C without exchanging the growth medium.

### i. Sendai virus rescue.

The amounts of SeV plasmids used for rescue were modified from previous work (27), as follows: 4 μg antigenomic rSeV^Fushimi^-EGFP construct, 1.44 μg T7-SeV^Fushimi^-N, 0.77 μg T7-SeV^Fushimi^-P, 0.07 μg T7-SeV^Fushimi^-L, 4 μg of T7opt, 5.5 μl PLUS reagent, and 8.9 μl Lipofectamine LTX. When the amounts of T7 construct or helper plasmids deviated from the standard amounts above, as in the experiments shown in Fig. 3 and 5, the amounts of Lipofectamine LTX and PLUS reagent were scaled to maintain the same ratios of these reagents to the total amount of DNA transfected.

### ii. Mumps virus rescue.

The amounts of MuV plasmids used were modified from those reported in previous work (29), as follows: 5 μg of antigenomic rMuV^JL5^-EGFP plasmid, 0.3 μg T7-MuV^JL5^-N, 0.1 μg T7-MuV^JL5^-P, 0.2 μg of T7-MuV^JL5^-L, 2 μg T7opt, 7.5 μl PLUS reagent, and 18.75 μl Lipofectamine LTX.

### iii. Newcastle disease virus rescue.

The amounts of NDV plasmids used were modified from those reported in previous work (30) as follows: 0.8 μg of antigenomic rNDV^LaSota^-EGFP plasmid, 0.4 μg T7-NDV^LaSota^-N, 0.2 μg T7-NDV^LaSota^-P, 0.2 μg of T7-NDV^LaSota^-L, 0.4 μg T7opt, 2.0 μl PLUS reagent, and 5.0 μl Lipofectamine LTX.

### iv. Measles virus rescue.

The amounts of rMeV^EdmonstonB^-EGFP plasmids used were modified from those of previous work (31) as follows: 5 μg of antigenomic rMeV^EdmonstonB^-EGFP plasmid, 1.2 μg T7-MeV-N, 1.2 μg T7-MeV-P, 0.4 μg of T7-MeV-L, 3 μg T7opt, 5.8 μl PLUS reagent, and 9.3 μl Lipofectamine LTX.

### v. Human parainfluenza type 3 virus rescue.

The amounts of rHPIV3^JS^-G.Luc-EGFP plasmids used for rescue were adapted from those of previous work with rSeV^Fushimi^-EGFP (27) as follows: 4 μg antigenomic rHPIV3^JS^-G.Luc-EGFP construct, 1.44 μg T7-HPIV3^JS^-N, 0.77 μg T7-HPIV3^JS^-P, 0.07 μg T7-HPIV3^JS^-L, 4 μg of T7opt, 5.5 μl PLUS reagent, and 8.9 μl Lipofectamine LTX.

### vi. Nipah virus rescue.

The amounts of NiV plasmids used for rescue were determined by homology with other systems, and the amounts of plasmid were adapted from those described in previous work (17) as follows: 7 μg antigenomic rNiV^Malaysia^-EGFP construct, 2.5 μg T7-NiV^Malaysia^-N, 1.6 μg T7-NiV^Malaysia^ −P, 0.8 μg T7-NiV^Malaysia^ −L, 2.0 μg of T7opt, 6.4 μl PLUS reagent, and 10.3 μl Lipofectamine LTX.

### Determinations of titers of viral supernatants.

Rescue cells were incubated at 37°C after transfection and monitored for the appearance of EGFP fluorescence. All titrations of virus stocks were performed on Vero cells in a 96-well format, with individual infection events identified via EGFP fluorescence at 24 h postinfection using an Acumen plate reader (TTP Labtech).

### Quantification of rescue efficiency.

Transfected cells were incubated at 37°C for 48 h, which is the earliest time point at which GFP can be reliably observed. Rescue cells were collected with trypsin, washed twice with Dulbecco’s phosphate-buffered saline (dPBS) plus 2% FBS, and fixed in dPBS plus 2% paraformaldehyde (PFA). To quantify rescue efficiency of rNiV^Malaysia^-EGFP, rescue cells were collected using trypsin, washed twice with dPBS plus 2% FBS, and fixed according to an approved protocol for biosafety level 4 (BSL-4) viruses. Briefly, infected cells were fixed by two successive rounds of incubation in dPBS plus 4% PFA overnight at room temperature, centrifugation at 1,250 rpm for 5 min, and replacement of the dPBS plus 4% PFA. For all reverse genetics systems, GFP-positive rescue events were quantified by flow cytometry using a FACSCanto II apparatus (BD Biosciences).

### Ribozyme cleavage assay.

The ribozyme cleavage assay was performed as previously described (17), with some modifications. BSR-T7/5 cells were transfected with 2 μg of antigenomic reverse genetics construct or 2 μg of empty pCAGGS in a 6-well format using Lipofectamine 2000 (Invitrogen) according to the manufacturer’s instructions. At 2 h posttransfection, cells were collected in TRIzol reagent (Invitrogen), and the RNA was extracted into water. RNA was treated with DNase (Invitrogen) at 1 mM MgCl_2_, treated with EDTA, and reverse transcribed with antigenome-specific primers using the SuperScript III first-strand synthesis system (Invitrogen) at 1 mM MgCl_2_. The SensiFAST SYBR and fluorescein kit (Bioline) was used to perform qPCR, with standard curves obtained via titration of the antigenomic constructs. Expression levels of antigenomic RNA were normalized using the expression level of hamster glyceraldehyde-3-phosphate dehydrogenase. The sequences of qPCR primers used in this assay are reported in Table S1 in the supplemental material.

10.1128/mSphere.00376-16.1 TABLE S1  Sequences of qPCR primers used in the ribozyme cleavage assay (sequences are listed for the qA and qB primer sets that were used in the ribozyme cleavage assay and transcript quantification; see Fig. 4A to C). Download TABLE S1, PDF file, 0.01 MB. Copyright © 2017 Beaty et al. 2017 Beaty et al. This content is distributed under the terms of the Creative Commons Attribution 4.0 International license.

### Accession number(s).

The optimized versions of all antigenomic plasmids and support plasmids have been sequenced and deposited in GenBank (accession numbers KY295909 to KY295932).
