# Supplementary material for: Efficient and Robust Paramyxoviridae Reverse Genetics Systems
Source: mSphere. 2017 Mar 29;2(2):e00376-16. doi: 10.1128/mSphere.00376-16 (PMC5371697; doi:10.1128/mSphere.00376-16)
Supplement: TABLE S1 [file sph002172258st1.pdf]

	Forward primer	Reverse primer
**rNDV^{LaSota}-EGFP (qA)**	5' - TCTAGACTCCGTCACCAAACA - 3'	5' - AGGAGCTGTTCGTACTCATCA - 3'
**rNDV^{LaSota}-EGFP (qB)**	5' - TGATGAGTACGAACAGCTCCT - 3'	5' - CCTGAGTGGTTTGTTGGCAT - 3'
**rMuV^{JL5}-EGFP (qA)**	5' - TCTAGACTCCGTCACCAAGG - 3'	5' - CCTCACCCCTGTCTTGAAGT - 3'
**rMuV^{JL5}-EGFP (qB)**	5' - ACTTCAAGACAGGGGTGAGG - 3'	5' - ATCTGAGCTTCGGGTGATCT - 3'
**rMeV^{EdmonstonB}-EGFP (qA)**	5' - TCTAGACTCCGTCACCAAACA - 3'	5' - GCTGAACTTGTGGCCGTTTA - 3'
**rMeV^{EdmonstonB}-EGFP (qB)**	5' - TAAACGGCCACAAGTTCAGC - 3'	5' - AAGTCGTGCTGCTTCATGTG - 3'
**rNiV^{Malaysia}-EGFP (qA)**	5' - TCTAGACTCCGTCACCAAACA - 3'	5' - GCTGAACTTGTGGCCGTTTA - 3'
**rNiV^{Malaysia}-EGFP (qB)**	5' - ACGTAAACGGCCACAAGTTC - 3'	5' - AAGTCGTGCTGCTTCATGTG - 3'
**rSeV^{Fushimi}-EGFP (qA)**	5' - TCTAGACTCCGTCACCAAACA - 3'	5' - GCACCTCCTCCCGACTTATT - 3'
**rSeV^{Fushimi}-EGFP (qB)**	5' - AATAAGTCGGGAGGAGGTGC - 3'	5' - AAGCAGAGAGACGAGGAACC - 3'
